# Population cluster data to assess the urban-rural split and electrification in Sub-Saharan Africa

**DOI:** 10.1038/s41597-021-00897-9

**Published:** 2021-04-23

**Authors:** Babak Khavari, Alexandros Korkovelos, Andreas Sahlberg, Mark Howells, Francesco Fuso Nerini

**Affiliations:** 1 Division of Energy Systems, KTH Royal Institute of Technology, Brinellvägen 68, 10044 Stockholm, Sweden; 2 The World Bank Group, Washington, DC 20433 USA; 3 Department of Geography and Environment, Loughborough University, Leicestershire, LE11 3TU UK; 4 Center for Environmental Policy, Imperial College, London, SW7 1NE UK; 5 RFF-CMCC European Institute on Economics and the Environment, Fondazione Centro Euro-Mediterraneo sui Cambiamenti Climatici, 20143 Milano, Italy

**Keywords:** Scientific data, Software, Developing world, Energy access

## Abstract

Human settlements are usually nucleated around manmade central points or distinctive natural features, forming clusters that vary in shape and size. However, population distribution in geo-sciences is often represented in the form of pixelated rasters. Rasters indicate population density at predefined spatial resolutions, but are unable to capture the actual shape or size of settlements. Here we suggest a methodology that translates high-resolution raster population data into vector-based population clusters. We use open-source data and develop an open-access algorithm tailored for low and middle-income countries with data scarcity issues. Each cluster includes unique characteristics indicating population, electrification rate and urban-rural categorization. Results are validated against national electrification rates provided by the World Bank and data from selected Demographic and Health Surveys (DHS). We find that our modeled national electrification rates are consistent with the rates reported by the World Bank, while the modeled urban/rural classification has 88% accuracy. By delineating settlements, this dataset can complement existing raster population data in studies such as energy planning, urban planning and disease response.

## Abstract (summary)

**Table:** 

Measurement(s)	electricity access • urban area • rural area
Technology Type(s)	digital curation
Factor Type(s)	Administrative boundaries • Population • Night-time lights
Sample Characteristic - Environment	populated place
Sample Characteristic - Location	Sub-Saharan Africa

Machine-accessible metadata file describing the reported data: 10.6084/m9.figshare.13516283

## Background & Summary

The 2030 Agenda for Sustainable Development has set the target of universal energy access^1^ (SDG 7.1). Scholarly^2–8^ and policy literature^9,10^ has indicated that this is a significant challenge, especially for rural communities of industrializing countries. The increase in electrification rate is unevenly distributed, and more than half of the population in Sub-Saharan Africa (SSA) still do not have access to electricity^10^. Electricity access inequality is present within the countries of the region, as urban electrification rates tend to be significantly higher than the rural ones^6,7,9–11^. Extending the grid to rural communities might not be economically attractive and therefore (as budgets are limited) these settlements often remain un-electrified^2,3^.

Geographic Information Systems (GIS) can inform the planning of future energy systems and facilitate rural electrification^12–15^. Energy modelling tools utilizing GIS can tailor solutions and actions to different parts of a study area more heterogeneously than traditional modelling frameworks. This is possible due to the spatial and temporal dimensions of GIS, which describe how different characteristics change across a study area based on location and time^16,17^. Furthermore, GIS and new high resolution satellite imagery can mitigate data gaps that often hamper energy planning in industrializing countries^16^. One example of this is night-time lights (NTL). NTL maps detect mostly anthropogenic lights, hence providing valuable insight into where there is electricity consumption during night-time hours. Previous studies highlight the relationship between the presence of NTL and electricity access and consumption^18–24^.

Knowing the spatial characteristics of population distribution is important in many applications such as, electrification planning^2,3,16,25–27^, urban planning^27–30^ and risk management^27,31–35^. *Falchetta et al*. produced and published datasets to assess electrification in SSA. They use NTL and population maps in order to assess where electrified people live and what the electricity consumption of these people are^36^. *Szabó et al*. and *Mentis et al*. carry out least-cost electrification studies for Africa and SSA respectively^15,25^. Both studies reach the conclusion that achieving universal electricity access requires large investments in off-grid systems. In both of the studies, demand is one of the main drivers behind the choice of technology and highly dependent on the population distribution.

Most of the available geospatial population datasets come in either raster format or as census data. Censuses have high level of accuracy if performed correctly, but the data collection is often time consuming and divided into different political units, leading to aggregated population counts^37–39^. When conducting spatial analysis it is often desirable to have population datasets in a uniform scale across the entire study area. Rasters can therefore be used in order to mitigate some of the shortcomings of census data. Furthermore, rasters have the ability to provide more timely estimates of population counts across larger areas in comparison to censuses^38,40^. However, rasters may fail to capture the area and shape that population settlements naturally have. Instead, they consist of pixelated areas, each pixel treated on its own, separated from adjacent cells^17^. This can have two implications; 1) different modelling results present themselves in the same settlement even in cases where these settlements are too small for this to be the actual case, and 2) the resolution of the population dataset can create biases (e.g., data represented at different spatial scales for the same study area might not generate consistent results^41^). This issue is labeled as the Modifiable Areal Unit Problem (MAUP)^42–45^. MAUP describes how statistical results change when geographical units change^46^. Gehlke and Biehl first discussed the importance of the choice of geographical units in spatial analysis in 1934 and Openshaw later expanded the concept in 1984^47,48^.

Vector-based population clusters can complement existing raster datasets. The Reiner Lemoine Institut previously generated consumer-clusters for Nigeria^49,50^. In these studies, they generate clusters using population maps and different nucleation points. The polygon nature of the clusters enables easy delineation of population settlements. In this publication, we develop a methodology to identify and generate vector-based population clusters using open-source GIS-layers. We also open-source the supporting code for higher transparency, reproducibility and replicability of the modelling process. The clustering methodology presented and published here has previously been used in *Korkovelos et al*. for application in Malawi^17^. Furthermore, it was previously used and developed as part of the Global Electrification Platform (https://electrifynow.energydata.info/) developed by the World Bank and in the World Energy Outlook of 2019^9^. With this publication, we further describe, refine and automate the process, including new attributes in regards to the urban-rural divide and an NTL-based electrification proxy for each cluster. As such, we generate, validate and publish open population “clusters” for 44 countries in SSA, for the first time.

## Methods

**Fig. 1:**
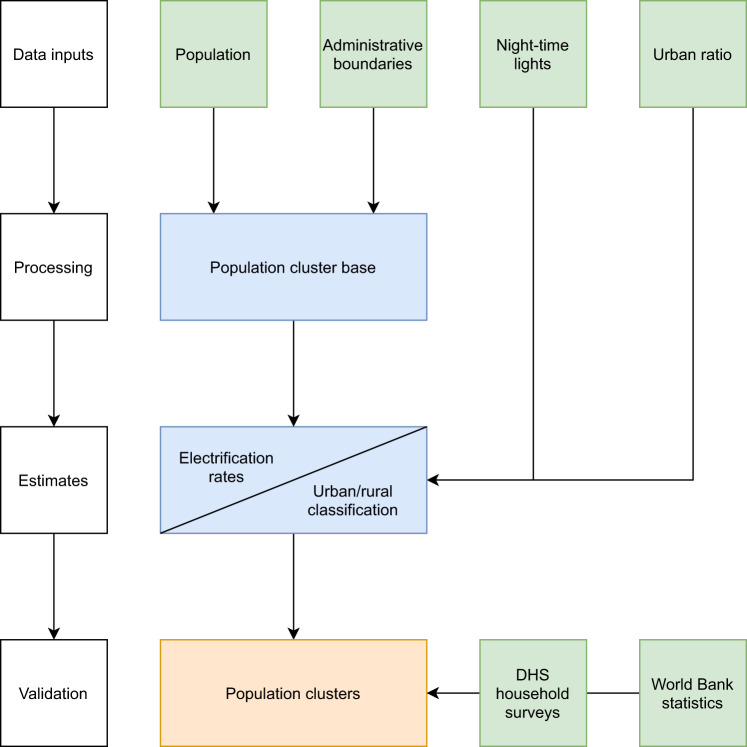
Simplified schematic of the clustering process and validation. Green: external data used in the process, these include GIS and non-GIS inputs. Blue: intermediate steps. Orange: Final output from the clustering algorithm.

Figure 1 presents a simplified overview of the methodology.

The implementation of the methodology presented in Fig. 1 is based on three initial datasets; a) gridded population (raster), b) night-time light intensity (raster) and c) administrative boundaries (vector). These datasets are available with different spatial and temporal resolutions. In Table 1, we present indicative open access data that we have tested in this paper.

**Table 1:** Data inputs selected and used in this paper for cluster generation and result validation.

Dataset	Name and source	Spatial resolution	Temporal coverage	Year used in analysis
Used for cluster generation				
Administrative boundaries	GADM Administrative Units v. 3.6^53^	—	2018	2018
Population	High Resolution Settlement Layer^54^	30 m	2003–2015 (country dependent, adjusted to match UN-estimates)	2003–2015 (adjusted for population in 2015)
	Global Human Settlement Layer^55^	250 m	1975, 1990, 2000, 2015	2015
	Unconstrained WorldPop^56^	100 m	2000–2020	2018
Night-time light	VIIRS DNB night-time lights^63^	450 m	2012–2020	2016

While the methodology described here is agnostic to input data it is important to note that certain datasets are not tested in this paper. Most notably, the CEISIN Gridded Population of the World version 4 (GPWv4)^51^ and LandScan^52^. Althgouh these datasets can be used in order to generate the clusters they have been ommited from this study. LandScan has been shown to preform well in urban areas while it is less accurate in rural regions. Furthermore, LandScan models ambient population rather than nighttime population, which is what we model with the clusters presented here^38^. For GPWv4 areal weighting is used in order to populate the grid cells in the population rasters. Using population censuses and administartive maps the population in each administartive unit is evenly divided into the cells that make up that specific unit. This methodology risks reporting considerably higher population values for rural areas that fall into large administrative units^38^.

### GIS data collection

#### Administrative boundaries

The administrative boundaries are used for two reasons; 1) delimit the population layer, ensuring that the population dataset that is used is on a national level and 2) limit the spatial extent of each cluster. In this analysis the disaggregated administrative boundaries from GADM v. 3.6 are used (level 1 or 2)^53^. The administrative units need to be in the form of polygons and in the WGS 84 coordinate reference system (EPSG:4326).

#### Population

The population density dataset is at the core of the clustering process and is in the form of a raster layer. By using a raster it is ensured that the clusters are all built by uniform cells with the same size and shape. Additionally, it is important for the raster to minimize the number of false positives. A false positive in this context is a cell that appears populated in the dataset, while being uninhabited in reality. False positives will lead to population settlements appearing larger than they actually are, as well as indicating population clusters where there are none. Likewise, it is important to minimize false negatives, buildings not existing in the dataset while doing so in satellite imagery. We selected and assessed three different population datasets; the High Resolution Settlement Layer (HRSL)^54^, the Global Human Settlement Layer (GHS-POP)^55^ and WorldPop (the unconstrained version)^56^.

Facebook Connectivity Lab and the Center for International Earth Science Information Network generates the HRSL datasets. Their methodology makes use of high-resolution tiles of satellite imagery to identify built-up areas. The buildings are then populated using the latest available population survey. In the case of SSA, the years of these surveys range from between 2003 and 2015 (the years presented in the estimates are however 2015)^38^. A drawback of this dataset is that it does not distinguish between different types of buildings and instead it populates all the buildings found in the satellite imagery^57^. As of the time of writing, HRSL covers most of Africa (with the exception of Somalia, Sudan and South Sudan), as well as 144 countries outside of Africa^54^.

The GHS-POP layer utilizes a similar method as HRSL, first identifying built areas using satellite imagery and then populating these areas with the GPWv4.10. The resulting dataset is available at 250 m or 1 km spatial resolution. The dataset covers four different epochs in time (1975, 1990, 2000 and 2015). An advantage of GHS-POP is the fact that the dataset has global coverage, well-documented methodologies and consistent time series that enables deeper temporal analysis^58^.

For the unconstrained WorldPop dataset, population census data from GPWv4 is reallocated at a finer scale using random forest regression techniques together with a number of different geospatial correlates (e.g. NTL, roads, land cover, built infrastructure etc.). Source codes and assumption used for generating the WorldPop datasets are publicly available and open-source. The unconstrained version of the dataset does not exclude areas without built infrastructure and therefore non-zero values can be found in regions that could be assumed uninhabited. The dataset is available on a global scale and on a yearly basis for the years of 2000-2020^38,59^.

The unconstrained WorldPop dataset gives the largest number of false positives as the national datasets completely cover the selected area. In September of 2020 a constrained version of WorldPop was released for sub-Saharan African countries^60^. This dataset uses the same methods as the unconstrained WorldPop dataset but similarly to HRSL and GHS-POP it uses a built-up layer to remove all cells that do not coincide with building footprints. This new version of WorldPop would presumably result in less false positives than its predecessor, but the dataset has not been tested as it exclusivly represents population for the year of 2020. Using this dataset for the clustering process would lead to problems during validation as DHS results and electricity access data for 2020 is currently not available. The GHS-POP and HRSL also give rise to false positives due to natural formations seen on satellite imagery sometimes being mistaken for buildings in the classification process. In some instances, there are false positives where no buildings are detected at all. If the satellites fail to identify any buildings in an administrative unit, HRSL and GHS-POP give similar results to GPWv4 (using areal weighing). Due to the satellite used for HRSL being of higher resolution, these instances are more prevalent to occur for GHS-POP^38^.

False negatives should also be avoided. Both HRSL and GHS-POP are subject to false negatives. This is the result of buildings not being found in different parts of the study area. Several studies have been conducted comparing GHS-POP to HRSL in regards to the built-up areas they find respectively^38,57,61,62^. Tiecke *et al*. conducted case studies for 18 countries comparing the performance of different geospatial population layers, amongst them HRSL and GHS-POP. As part of their study they also assess the recall values for GHS-POP and HRSL in urban and rural settings against a manually labeled area in Malawi. In urban areas both datasets perform well (with recall values of 0.99 and 0.83 for HRSL and GHS-POP respectively), but for rural areas HRSL outperformed GHS-POP (recall values of 0.84 and 0.04 respectively). This suggests that HRSL is the better option in rural settings^57^. Engstrom *et al*. propose a bottom-up approach to generate population estimates and apply it to the case of Sri Lanka in order to predict population counts in non-surveyed areas and in between survey years. As part of the study, they compare GHS, HRSL and WorldPop (the unconstrained 2015 version) to their bottom-up approach. Their analysis show that HRSL and WorldPop are the only two layers that correlate fairly well with the census data used. They attribute this to the satellite imagery used for HRSL and WorldPop being of higher resolution than for the other datasets^62^.

Based on the available literature and methodologies used to generate the different population layers, HRSL is chosen as the primary population map in this paper. In cases where HRSL is not available (for Sudan, South Sudan and Somalia), GHS-POP is used.

#### Night-time lights

There are multiple sources of night-time light imagery that can be used. The optimal results will however be achieved by using a dataset cleaned from noise. Noise in this context refers to light being seen on the maps without being emitted from a stable source (e.g. lights being emitted from boats, fires, gas-flaring etc. or because of blooming effects around large cities). The night-time light maps used for these clusters are the ones generated from the Visible Infrared Imaging Radiometer Suite (VIIRS) Day/Night Band (DNB). The VIIRS dataset is of global coverage and in the following study the yearly composite of 2016, the latest yearly composite available at the time of writing, has been used^63^.

### Non-GIS inputs

Apart from GIS data the population in the study year (used to do a simple calibration of the GIS population), the national urban ratio in the study year (used to determine urban, peri-urban and rural clusters), the name of the study area and the coordinate reference system used for projecting the clusters are needed. The calibration of population is important in order to ensure that the total population in the clusters is in line with the year chosen by the user. The calibration is done by multiplying the same factor to all clusters. For the clusters produced with this paper, we have chosen 2016 as the year (since this match the year of the NTL-map). Population values and urban ratios are from the United Nations Department of Economic and Social Affairs^64,65^.

The coordinate reference system used in order to project the clusters will determine the unit of the area measurements. Therefore, the unit of the coordinate reference system has to be linear. We use World Mercator (EPSG:3395) for the clusters produced with this paper.

### Data transformation

Pre-processing the GIS-layers is necessary before the clustering algorithm is used. The pre-processing steps are:

- Ensure that all datasets have the same coordinate system. Having different coordinate systems might lead to errors during the processing. Most GIS-data come in the coordinate system WGS 84 (EPSG:4326) and for the clusters produced with this paper all datasets have been projected to this coordinate system before the clustering process starts.
- Ensure all features in the polygon administrative map are valid. For example, this can be done using “Fix geometries” in QGIS or “Repair geometry” in ArcGIS. If the features of a vector dataset is not valid, certain operations such as clipping rasters will crash.
- The raster layers have to be in TIFF-format. This can be ensured by e.g. clipping the raster to the area of interest in QGIS or ArcGIS and then export it as a.TIF-file.

### Cluster generation

The clustering process can be split into three separate workflows:

1. Generating the cluster base
2. Generating an indicative measurement of electrification rate in each cluster
3. Classifying clusters as either urban, peri-urban or rural

An open source repository for cluster generation is available at https://github.com/babakkhavari/Clustering. Below descriptions of the three separate workflows follow.

#### Generating population clusters

In the first workflow the base of the clusters is generated. This is done by using two GIS-layers (population raster and administrative boundary polygons), a population threshold (integer entered by the user) and the population in the start year (integer entered by the user). The population layer is clipped by the administrative boundaries, upon which low-density cells are removed. Low-density cells are defined as all cells with lower population density than the threshold entered by the user. This enables the use of population layers with high number of false negatives such as the unconstrained WorldPop data. Following this step, the population is calibrated using the population in the start year by multiplying all remaining cells with the same factor.

**Fig. 2:**
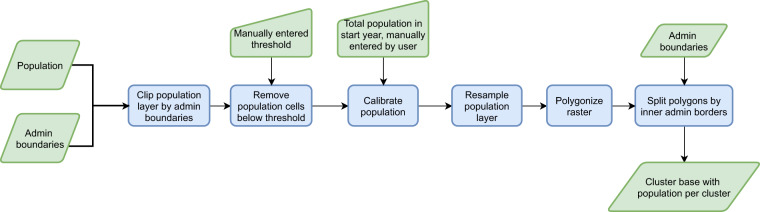
Flowchart describing the process used for generating the cluster base.

After removal of low-density population cells and calibration, the resulting layer is polygonised. All cells that are adjacent to one-another (8-connected neighbors) are merged into one cluster. The last step in this workflow is to split the clusters based on the inner borders of the administrative boundaries. This is done in order to enable local leaders, policy makers and researchers to focus on the population in certain regions, departments and communes. If one would wish to skip this step, admin boundaries of level 0 (national borders) can be used. Figure 2 shows the framework used in this workflow.

#### Electrification rate

We determine the electrification rate in each cluster by first delimiting areas with visible night-time lights. Then we sum the population in these areas and add them to their corresponding cluster. Every cluster also indicates the maximum night-time light intensity detected in it. This enables filtering of electrified population deemed to live in areas with too low night-time light intensities.

#### Urban distribution

Historically, there has not been a globally accepted method for classifying population settlements into urban and rural^66^. Some countries have used population density thresholds with densely populated areas defined as urban^67^. Other countries have used population size. By first defining what can be considered one single population settlement, each settlement can then be classified as either urban or rural based on the number of inhabitants^67^. Using nationally defined values may lead to certain countries having a far higher threshold than others, and comparing one country to another may therefore be problematic.

In recent times, official efforts have been made towards finding a unified way of representing urbanization. One of the more widely used methods is the *Degree of Urbanisation* by Eurostat. Using population datasets, settlements globally are classified as either urban centres (urban), urban clusters (peri-urban) or rural. These settlements are defined using one threshold for settlement size and one for population density. We present these thresholds in Table 2^67^.

**Table 2:** Thresholds used by Eurostat when classifying settlements.

	Urban	Peri-urban	Rural
Density threshold	1,500 people per sq. km	300 people per sq. km	< 300 people per sq. km
Size threshold	50,000 inhabitants in settlement	5,000 inhabitants in settlement	< 5,000 people in settlement

These thresholds generate a global dataset classifying all regions of all countries. However, the split between urban and rural using this methodology does not fit with the national splits presented by different countries. Densely populated countries tend to have higher thresholds while sparsely populated countries have lower ones^67^.

**Fig. 3:**
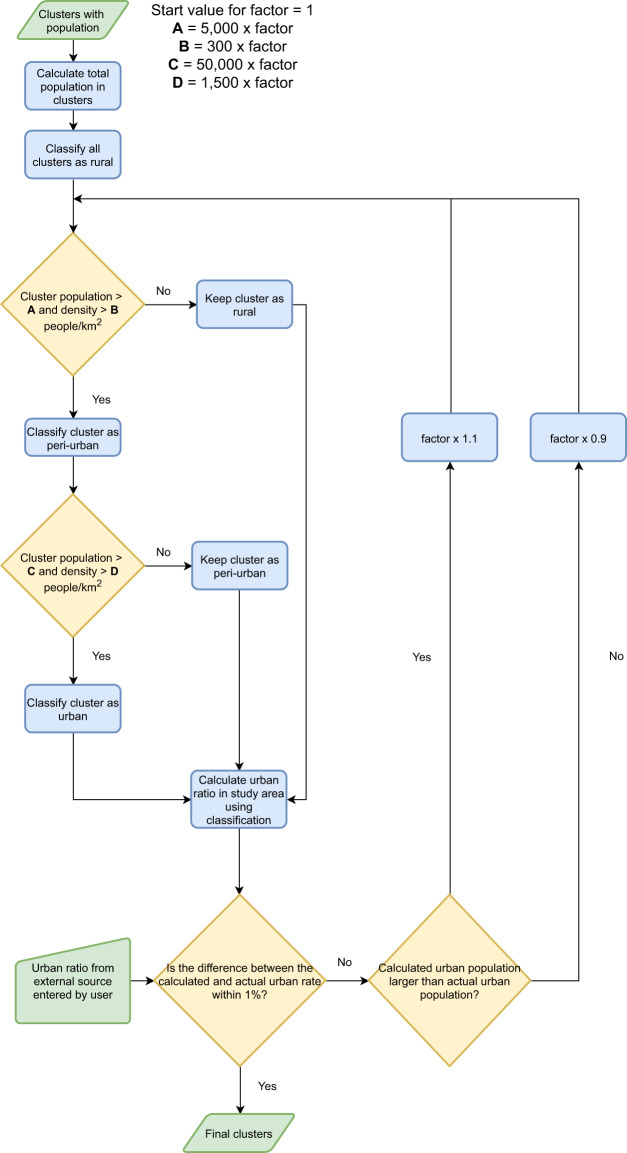
Flowchart describing the process used for urban classification of clusters.

When generating the national population clusters we want the national urban ratio to be equivalent to official statistics. Therefore, we use the values above only as common starting values for the classification. Through an iterative process (Fig. 3), we sum the urban population and determine the urban ratio. If the urban ratio is too large compared to the value entered by the user, the thresholds are increased. Similarly, if the urban ratio is lower than the national value we decrease the thresholds. Peri-urban settlements are defined as the transition zones between urban and rural areas.

### Limitations

The methods used to generate the population clusters are agnostic to the datasets used. Since the population dataset provides the base for the population clusters future research should test and validate the methods presented here with other population datasets e.g. the GPWv4^51^, the LandScan Global Population^52^ and the constrained version of WorldPop^60^.

Raster population data is based on statistical downscaling of census data^39,68,69^ This process by itself induces uncertainty to the final raster products. Also, national censuses may follow certain protocols that can lead to inconsistencies. For example, omitting certain groups of the population e.g. nomads, homeless and displaced people due to them not having a permanent residence, people in institutions and people living in areas considered security risks. Carr-Hill estimates that globally between 300 and 350 million people are affected by this^68^. The above can induce compounding uncertainty to the input population rasters, which is ultimately propagated to our clustering result.

Moreover, raster datasets can store unique information in each pixel. This creates the ability to generate heterogeneous maps with high levels of detail. However, when the raster datasets are aggregated to population clusters, this detail can get lost due to multiple cells being merged into single units. This complicates analysis on sub-settlement level and may give rise to modifiable areal unit problems (MAUP). MAUP is a well-known phenomenon in spatial analysis related to the scale at which geographical data is represented. Data represented at different scales might produce inconsistent modelling results for the same study area^41^. This is an issue for rasters when changing resolutions, but also when aggregating raster cells to polygon clusters. An example of this is renewable energy resources. In a raster-based analysis the resources in each pixel are treated cell by cell, but in vector settlements the data will have to be generalised, sometimes across large areas (e.g. instead of extracting wind velocity to each cell, the average value across the cluster is used). As the clusters get larger, these issues become more prominent. Future research should examine the effects that MAUP has on the clusters presented here as well as how this may impact subsequent analyses based on our clusters e.g. electrification planning. This is important to asses, as the effects of MAUP can potentially lead to compounding errors such as propagation and cascading. These types of errors can lead to the results of the GIS analysis becoming inaccurate.

## Data Records

The clusters are available through a permanent Mendeley database (https://data.mendeley.com/datasets/z9zfhzk8cr/6)^70^. The data files are in the form of GIS-compatible vector polygons (ESRI Shapefiles). The datasets are available on national level representing 44 countries (mainland SSA and Madagascar). Each dataset contain the following information:

1. **id** – A unique identifier for the cluster.
2. **Country**
3. **Population** – Headcount of people in each cluster for the base year
4. **NightLight** – Maximum luminance detected in each cluster
5. **ElecPop** – The number of people in the cluster who live in areas with visible night-time light, used as a proxy for electrification rate
6. **Area** – Area of the cluster in sq.km.
7. **IsUrban** – Discrete identifier, signifying whether a settlement is urban (2), peri-urban (1) or rural (0).

The datasets are available with a Creative Commons Attribution 4.0 International license (CC BY 4.0).

## Technical Validation

### Urban distribution

To determine the validity of the urban classification, we use the Demographic and Health Surveys (DHS) for 22 countries conducted between 2014 and 2018^71^. The surveys include coordinates of settlements as well as their urban/rural status. These surveys are developed to be representative on a national scale and usually have a sample size of between 5,000 and 30,000 households. Processing of survey data across all countries indicate 3,406 urban and 6,513 rural settlements. Our analysis identifies 6,142 urban settlements across these 22 countries. Supplementary Fig. 1 shows urban, peri-urban and rural settlements in coastal regions of Ghana, Togo, Benin and western Nigeria in red, orange and green respectively. Note that certain urban regions in our analysis are split into more than one cluster due to us using disaggregated administrative maps. If administrative level 0 is used instead the number of urban settlements are 4,557. To evaluate our methodology we use a set of performance diagnostics, as presented in the following paragraphs.

We conduct the evaluation using a confusion matrix, as this is a powerful tool when assessing the results of classification problems such as our urban-rural classification. A confusion matrix consists of true positives (TP), true negatives (TN), false positives (FP) and false negatives (FN). We get a TP every time a cluster is correctly predicting an urban settlement from the DHS data, while a TN is correctly identifying rural settlements from the DHS data. An FP occurs when the clusters misclassifies a rural DHS-settlement as urban and an FN is the opposite, an urban DHS-settlement misclassified as rural.

To assess the success of the classification method we use accuracy as determined based on Eq. 1.

1 $$\frac{TP+TN}{TP+FP+TN+FN}=Accuracy$$

This gives a measurement for how often the classification is correct. Across all countries, 27% of the observations are TP, 61% are TN. This puts the total accuracy at 88%. On a national level, Burkina Faso has the highest accuracy (95%), while Kenya has the lowest (71%). Accuracy is a good measurement when avoiding false positives and negatives are of equal importance. There is however a risk that accuracy gets skewed by class imbalances. Due to class imbalance existing in the survey data (only 33% of all DHS observations are urban), we also use recall and precision to assess the results of our urban classification.

Recall, in this case, is a measurement of how often we correctly manage to identify urban areas when dealing with urban areas (Eq. 2). Precision is a measurement of how large portion of our urban areas that are actual urban areas (Eq. 3).

2 $$\frac{TP}{TP+FN}=Recall$$

3 $$\frac{TP}{TP+FP}=Precision$$

For the 22 cases, recall runs between 0.47 and 0.98, while precision runs between 0.57 and 1. Due to the definition of these two measurements, they cannot be maximized simultaneously. Due to us valuing FPs and FNs as equally bad, we want a balance between recall and precision. Therefore, we use the Jaccard Index (IoU) (Eq. 4).

4 $$\frac{TP}{TP+FN+FP}=Jaccard\,Index$$

The IoU can provide a more accurate performance metric than accuracy in datasets with class imbalances by omitting the TN. If a classification algorithm gives an IoU above 0.5 the results are considered to be of good quality. This score ranges from 0.44 for Kenya to 0.85 for Angola.

Two countries, Rwanda and Kenya, have an IoU lower than 0.5 (0.48 and 0.44 respectively). This is due to a disproportionately large numbers of false negatives. Using population density and population size for the urban classification in Kenya gives 54 true positives and 57 false negatives, while in Rwanda the same numbers are 69 and 44. Rwanda is one of the most densely populated countries in SSA and Kenya – even though nationally not densely populated – has a large majority of its population living in the southern regions of the country^72^. This implies that when population density increase, so does the risk of the density based urban-rural classification faltering. See detailed results for all countries in Supplementary Table 1.

### Electrification rate

**Fig. 4:**
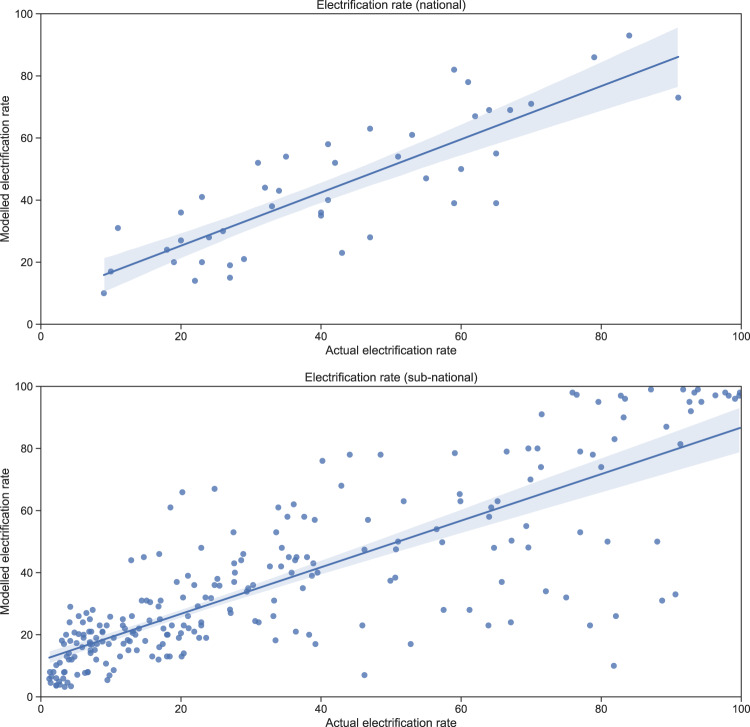
(Top) Linear fit model for national electrification rates based on World Bank data. (Bottom) Linear fit model for sub-national electrification rates from DHS STATcompiler.

For validating the electrification rates on national and sub-national levels, the linear fit between survey and modelled data is examined. National data is from the World Bank while the sub-national electrification rates are from DHS STATcompiler. For the World Bank data we use the reference year of 2016 as this matches the year of our NTL data. The DHS data we use is from between 2014 and 2018 depending on country. All settlements with visible night-time lights are considered electrified. The linear fit model indicates a coefficient of determination of R^2^ = 0.68 on the national level and 0.66 on sub-national level (see Fig. 4). See Supplementary Tables 2 and 3 for all countries’ electrification rates according to the World Bank and the sub-national rates as reported by DHS STATcompiler respectively.

The method provides satisfactory results for Mali, Equatorial Guinea, Gambia, Mauritania, Namibia, Zimbabwe and Eswatini. This is in-line with what Falchetta *et al*. reports, as their method also performs well in the aforementioned countries^36^. The largest underestimations can be seen in Nigeria (−20%), Ethiopia (−20%), Eritrea (−19%) and Kenya (−17%). The methodology used by Falchetta *et al*. gives an underestimation of 26.8% for Ethiopia, for Nigeria and Kenya their predictions are closer to the national statistics^36^.

Underestimations could be due to the night-time lights being best suited for detection of outdoor lighting. In order for indoor lighting to be detected on night-time light maps it would require considerable light leakage or high-intensity lighting^18,22^. This could lead to large underestimations of electrification rates in rural areas as the electricity consumption in these households may be smaller than what the satellites can detect. Many households that consume small quantities of electricity rely on off-grid systems, as they are more cost-effective in these settings^9,10^. An estimated 8.7 million people in Kenya get electricity from off-grid solar solutions^72^ and off-grid electrification options in Ethiopia powers 12% of the population^73^. This points towards the population living in areas with visible night-time lights being more reflective of population electrified by the national grid as these areas tend to have higher consumption^36,74^. Additionally, the VIIRS satellite has an overpass at around 1:30 am^75^. During this time most households have less light sources on, which further decreases the chance of light leakage.

Cases of considerable overestimation can be seen in Djibouti (+30%), Zambia (+27%) and Guinea Bissau (+26%). Overestimations of this magnitude could be due to the NTL maps detecting outdoor lights in areas where there is no residential electricity consumption^76,77^. The presence of outdoor lighting does not necessarily entail the existence of electrified households. It is also important to note that we assume that every person living in the entire lit area is electrified which is most likely not the case. Furthermore, we do not use any threshold in the NTL maps for these results (this option is however available in the algorithm published with this paper). Every cluster includes a column for the maximum luminosity and the user can utilize this column to filter out clusters deemed to have too low NTL values. The discrepancies in results between this study and the study conducted by Falchetta *et al*. most likely stem from the threshold of 0.25 *μW* · *cm*^−2^ · *sr*^−1^ that they apply to the NTL maps of 2016^36^. The stable light maps have values lower than this, which would entail our study finding more people living in areas with visible night-time lights. To estimate electrification rates more precisely, the authors recommend combining these clusters with information regarding electricity infrastructure. Supplementary Fig. 2 shows binary electrification status in coastal regions of Ghana, Togo, Benin and western Nigeria. Blue represents clusters with electricity accesses and yellow represents clusters that are not electrified.

## Usage Notes

The following data repository https://data.mendeley.com/datasets/z9zfhzk8cr/6^70^ includes ESRI Shapefiles in the EPSG:4326 coordinate reference system. All datasets are available on national scale. The information included in these clusters are id, country name, population, urban-rural classification, maximum nighttime light intensity and population living in areas with visible night-time lights.

As noted previously the data describes population settlements, which are key in many applications such as, but not limited to, electrification planning, urban planning and disaster response. In disaster response, the dataset can help researchers and policy makers to better understand the effects seen on different population settlements after a disaster, which can assist in mitigation and response efforts. Together with the data regarding electrification rate and urban-rural divide, these datasets can provide a starting point in electrification studies and electrification inequality assessments. These vector-based population clusters capture the geometries of settlements more detailed than raster cells, which helps in electrification planning by e.g. enabling assessments of power network designs or determining distances to different types of infrastructure more accurately. These clusters are not meant to substitute existing raster data, but rather complement them.

## Supplementary information

Supplementary Material

## Data Availability

The latest version of the code is available at https://github.com/babakkhavari/Clustering (GNU General Public License v3.0). The code is Python-based and runs in Jupyter Notebook. The code repository includes instructions for how to install and run the algorithm as well as a country example displaying the necessary inputs and expected outputs. The datasets published with this paper were ran using Python 3.6 and the packages listed in the full_project.yml file uploaded to the repository.
