# Supplementary material for: Population cluster data to assess the urban-rural split and electrification in Sub-Saharan Africa
Source: Sci Data. 2021 Apr 23;8:117. doi: 10.1038/s41597-021-00897-9 (PMC8065116; doi:10.1038/s41597-021-00897-9)
Supplement: Supplementary file 1 — Supplementary Material [file 41597_2021_897_MOESM1_ESM.docx]

Supplementary material for Population cluster data to assess the urban-rural split and electrification in Sub-Saharan Africa

### Authors

Babak Khavari^1^, Alexandros Korkovelos^1, 2^, Andreas Sahlberg^1^, Mark Howells^3, 4^, Francesco Fuso Nerini^1, 5^

**Affiliations**

1. Division of Energy Systems, KTH Royal Institute of Technology, Brinellvägen 68, 10044 Stockholm, Sweden

2. The World Bank Group, Washington, DC 20433, USA

3. Department of Geography and Environment, Loughborough University, Leicestershire LE11 3TU, UK

4. Center for Environmental Policy, Imperial College, London SW7 1NE, UK

5. RFF-CMCC European Institute on Economics and the Environment, Fondazione Centro Euro-Mediterraneo sui Cambiamenti Climatici, 20143 Milano, Italy

Corresponding author: Babak Khavari ([khavari@kth.se](mailto:khavari@kth.se))

# List of tables

[**Table 1.** Confusion matrix results for the 22 countries examined with DHS data. 2](#_Toc61695057)

[**Table 2**. National electrification rates for 2016 as reported by the World Bank and modelled electrification rates in the clusters 3](#_Toc61695058)

[**Table 3.** Sub-national electrification rates as reported by the DHS surveys in comparison to modelled electrification rates 4](#_Toc61695059)

# List of Figures

[**Fig. 1.** Urban, peri-urban and rural settlements in coastal regions of Ghana, Togo, Benin and western Nigeria in red, orange and green respectively 2](#_Toc60749148)

[**Fig. 2.** Binary electrification status in coastal regions of Ghana, Togo, Benin and western Nigeria. Blue represents clusters with electricity accesses and yellow represents clusters that are not electrified. 10](#_Toc60749149)

## Figure 1.


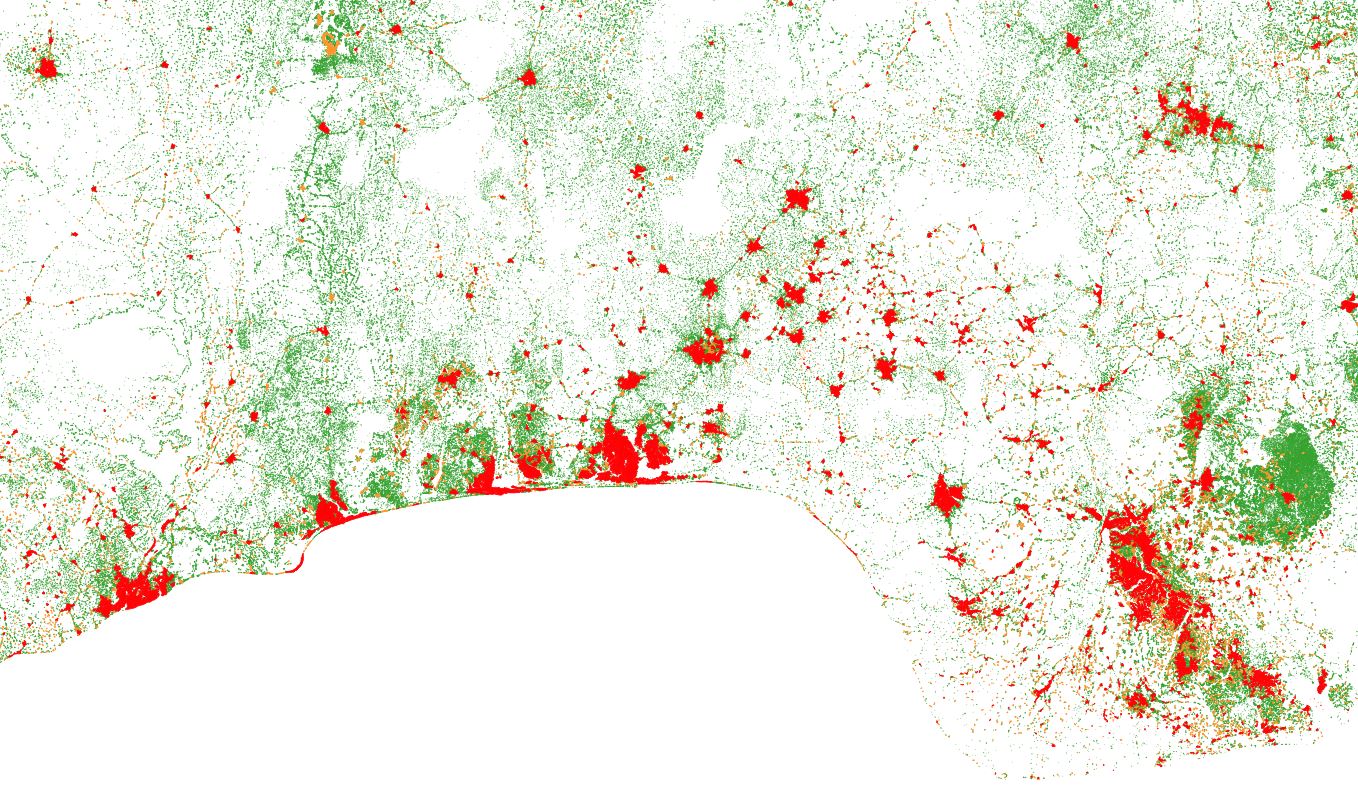


**Fig. 1.** Urban, peri-urban and rural settlements in coastal regions of Ghana, Togo, Benin and western Nigeria in red, orange and green respectively

## Table 1.

**Table 1.** Confusion matrix results for the 22 countries examined with DHS data.

| Country | Year | TP | TN | FP | FN | Acc | Rec | Prec | F1-score | IOU |
| --- | --- | --- | --- | --- | --- | --- | --- | --- | --- | --- |
| Angola | 2015 | 339 | 226 | 54 | 6 | 0.90 | 0.98 | 0.86 | 0.92 | 0.85 |
| Benin | 2017 | 172 | 253 | 44 | 71 | 0.79 | 0.71 | 0.80 | 0.75 | 0.60 |
| Burkina Faso | 2017 | 46 | 186 | 7 | 6 | 0.95 | 0.88 | 0.87 | 0.88 | 0.78 |
| Burundi | 2016 | 63 | 438 | 9 | 42 | 0.91 | 0.60 | 0.88 | 0.71 | 0.55 |
| Chad | 2015 | 124 | 456 | 5 | 39 | 0.93 | 0.76 | 0.96 | 0.85 | 0.74 |
| Ethiopia | 2016 | 175 | 403 | 17 | 27 | 0.93 | 0.87 | 0.91 | 0.89 | 0.80 |
| Ghana | 2016 | 86 | 83 | 13 | 10 | 0.88 | 0.90 | 0.87 | 0.88 | 0.79 |
| Guinea | 2018 | 121 | 255 | 8 | 17 | 0.94 | 0.88 | 0.94 | 0.91 | 0.83 |
| Kenya | 2015 | 54 | 121 | 9 | 61 | 0.71 | 0.47 | 0.86 | 0.61 | 0.44 |
| Liberia | 2016 | 50 | 80 | 0 | 20 | 0.87 | 0.71 | 1.00 | 0.83 | 0.71 |
| Madagascar | 2016 | 54 | 254 | 41 | 9 | 0.86 | 0.86 | 0.57 | 0.68 | 0.52 |
| Malawi | 2015 | 118 | 669 | 8 | 55 | 0.93 | 0.68 | 0.94 | 0.79 | 0.65 |
| Mali | 2015 | 44 | 120 | 12 | 1 | 0.93 | 0.98 | 0.79 | 0.87 | 0.77 |
| Nigeria | 2015 | 110 | 164 | 22 | 26 | 0.85 | 0.81 | 0.83 | 0.82 | 0.70 |
| Rwanda | 2015 | 69 | 348 | 31 | 44 | 0.85 | 0.61 | 0.69 | 0.65 | 0.48 |
| Senegal | 2016 | 70 | 116 | 14 | 14 | 0.87 | 0.83 | 0.83 | 0.83 | 0.71 |
| Sierra Leone | 2016 | 84 | 231 | 6 | 15 | 0.94 | 0.85 | 0.93 | 0.89 | 0.80 |
| South Africa | 2017 | 365 | 223 | 59 | 99 | 0.79 | 0.79 | 0.86 | 0.82 | 0.70 |
| Tanzania | 2015 | 153 | 398 | 30 | 27 | 0.91 | 0.85 | 0.84 | 0.84 | 0.73 |
| Uganda | 2016 | 119 | 482 | 45 | 39 | 0.88 | 0.75 | 0.73 | 0.74 | 0.59 |
| Zambia | 2018 | 170 | 316 | 23 | 26 | 0.91 | 0.87 | 0.88 | 0.87 | 0.78 |
| Zimbabwe | 2015 | 136 | 221 | 13 | 30 | 0.89 | 0.82 | 0.91 | 0.86 | 0.76 |

## Table 2.

**Table 2**. National electrification rates for 2016 as reported by the World Bank and modelled electrification rates in the clusters

| Country | Actual electrification rate as reported by the World Bank (%) | Modelled electrification rate (%) |
| --- | --- | --- |
| Angola | 41 | 58 |
| Benin | 42 | 52 |
| Botswana | 61 | 78 |
| Burkina Faso | 19 | 28 |
| Burundi | 9 | 10 |
| Cameroon | 60 | 50 |
| CAR | 14 | 15 |
| Chad | 9 | 17 |
| Djibouti | 52 | 82 |
| DRC | 17 | 24 |
| Equatorial Guinea | 68 | 69 |
| Eritrea | 47 | 28 |
| Eswatini | 66 | 71 |
| Ethiopia | 43 | 23 |
| Gabon | 91 | 72 |
| Gambia | 48 | 47 |
| Ghana | 79 | 86 |
| Guinea | 34 | 43 |
| Guinea Bissau | 15 | 41 |
| Ivory coast | 64 | 69 |
| Kenya | 56 | 39 |
| Lesotho | 30 | 44 |
| Liberia | 20 | 36 |
| Madagascar | 23 | 20 |
| Malawi | 11 | 31 |
| Mali | 25 | 35 |
| Mauritania | 41 | 40 |
| Mozambique | 26 | 30 |
| Namibia | 52 | 54 |
| Niger | 16 | 20 |
| Nigeria | 59 | 39 |
| Rep Con | 57 | 67 |
| Rwanda | 29 | 21 |
| Senegal | 65 | 55 |
| Sierra Leone | 20 | 27 |
| Somalia | 30 | 52 |
| South Africa | 84 | 93 |
| South Sudan | 9 | 14 |
| Sudan | 39 | 61 |
| Tanzania | 33 | 39 |
| Togo | 47 | 63 |
| Uganda | 27 | 19 |
| Zambia | 27 | 54 |
| Zimbabwe | 38 | 36 |

## Table 3.

**Table 3.** Sub-national electrification rates as reported by the DHS surveys in comparison to modelled electrification rates

| Country | Sub-region | Percentage of population electrified as reported in DHS survey (%) | Modelled electrification rate (%) |
| --- | --- | --- | --- |
| Angola | Bengo | 35.8 | 40 |
|  | Benguela | 38 | 45 |
|  | Bié | 6.5 | 27 |
|  | Cabinda | 66.5 | 79 |
|  | Cuando Cubango | 33.6 | 53 |
|  | Cuanza Norte | 35.2 | 58 |
|  | Cuanza Sul | 21 | 26 |
|  | Cunene | 15.9 | 13 |
|  | Huíla | 33.3 | 31 |
|  | Huambo | 23.5 | 32 |
|  | Luanda | 79.6 | 95 |
|  | Lunda Norte | 27.5 | 53 |
|  | Lunda Sul | 24.8 | 67 |
|  | Malanje | 36.5 | 45 |
|  | Moxico | 21 | 39 |
|  | Namibe | 51.8 | 63 |
|  | Uíge | 21.9 | 36 |
|  | Zaire | 36.1 | 62 |
| Benin | Atacora | 20.4 | 23 |
|  | Donga | 36.3 | 44 |
|  | Atlantique | 42.9 | 68 |
|  | Littoral (Cotonou) | 87.1 | 99 |
|  | Borgou | 24.8 | 36 |
|  | Alibori | 23.6 | 19 |
|  | Mono | 33.9 | 61 |
|  | Couffo | 27.1 | 27 |
|  | Ouémé | 48.5 | 78 |
|  | Plateau | 25.2 | 38 |
|  | Zou | 34.4 | 48 |
|  | Collines | 32.7 | 42 |
| Burkina Faso | Boucle du Mouhoun | 4.8 | 20.7 |
|  | Cascades | 18.1 | 20 |
|  | Centre | 59.1 | 78.5 |
|  | Centre-Est | 9.9 | 25.8 |
|  | Centre-Nord | 7 | 17.6 |
|  | Centre-Ouest | 7.2 | 21 |
|  | Centre-Sud | 8.8 | 17.8 |
|  | Est | 5.1 | 17.3 |
|  | Hauts-Bassins | 27.7 | 40 |
|  | Nord | 8.8 | 20.8 |
|  | Plateau Central | 5.4 | 20.2 |
|  | Sahel | 9.3 | 10.7 |
|  | Sud-Ouest | 4.8 | 12.9 |
| Burundi | Bubanza | 7.1 | 14.1 |
|  | Bujumbura Mairie | 69.6 | 48.1 |
|  | Bujumbura Rural | 9.5 | 5.4 |
|  | Bururi | 3.3 | 7.9 |
|  | Cankuzo | 2.2 | 3.7 |
|  | Cibitoke | 9.8 | 6.9 |
|  | Gitega | 7.7 | 15.1 |
|  | Karusi | 1.5 | 6.5 |
|  | Kayanza | 6.3 | 7.7 |
|  | Kirundo | 2.2 | 3.6 |
|  | Makamba | 3.8 | 4.6 |
|  | Muramvya | 2.2 | 10.2 |
|  | Muyinga | 1.8 | 8 |
|  | Mwaro | 2.6 | 4.9 |
|  | Ngozi | 7.3 | 16.9 |
|  | Rumonge | 10.4 | 8.6 |
|  | Rutana | 4.3 | 3.4 |
|  | Ruyigi | 3.4 | 3.2 |
| Ethiopia | Addis Abeba | 99.8 | 98 |
|  | Affar | 22.6 | 19 |
|  | Amhara | 18.4 | 13 |
|  | Ben-Gumz | 18 | 13 |
|  | Dire Dawa | 64.7 | 48 |
|  | Gambela | 38.3 | 20 |
|  | Harari | 75 | 32 |
|  | Oromiya | 16.9 | 16 |
|  | SNNP | 13.7 | 15 |
|  | Somali | 13.5 | 20 |
|  | Tigray | 31.1 | 24 |
| Ghana | Ashanti | 83.2 | 90 |
|  | Brong-Ahafo | 77 | 79 |
|  | Central | 71.5 | 91 |
|  | Eastern | 70.9 | 80 |
|  | Greater Accra | 93.3 | 98 |
|  | Northern | 59.9 | 63 |
|  | Upper East | 46.7 | 57 |
|  | Upper West | 65.2 | 63 |
|  | Western | 78.8 | 78 |
|  | Volta | 81.9 | 83 |
| Kenya | Coastal Endemic | 37.6 | 58 |
|  | Highland Epidemic | 18.7 | 23 |
|  | Lake Endemic | 17 | 25 |
|  | Low Risk | 56.5 | 54 |
|  | Semi-Arid/Seasonal Risk | 22.9 | 23 |
| Malawi | Chitipa | 7.9 | 12 |
|  | Karonga | 8.4 | 23 |
|  | Mzimba | 4.1 | 24 |
|  | Rumphi | 14.9 | 18 |
|  | Nkhata Bay | 12.5 | 15 |
|  | Dedza | 1.3 | 8 |
|  | Dowa | 4 | 12 |
|  | Kasungu | 5.9 | 16 |
|  | Lilongwe | 16.9 | 46 |
|  | Mchinji | 3.3 | 17 |
|  | Nkhotakota | 9.4 | 23 |
|  | Ntcheu | 4.4 | 12 |
|  | Ntchisi | 6.7 | 8 |
|  | Salima | 6 | 24 |
|  | Balaka | 6.1 | 19 |
|  | Blantyre | 40.2 | 76 |
|  | Chikwawa | 5.4 | 26 |
|  | Chiradzulu | 4.2 | 18 |
|  | Machinga | 4 | 14 |
|  | Mangochi | 7 | 25 |
|  | Mulanje | 7.9 | 19 |
|  | Mwanza | 13 | 26 |
|  | Neno | 3.7 | 13 |
|  | Nsanje | 7.4 | 28 |
|  | Phalombe | 2.7 | 11 |
|  | Thyolo | 6.9 | 21 |
|  | Zomba | 11.7 | 25 |
| Nigeria | Abia | 90.6 | 33 |
|  | Adamawa | 14.4 | 31 |
|  | Akwa Ibom | 78.4 | 23 |
|  | Anambra | 88.7 | 31 |
|  | Bauchi | 39.2 | 17 |
|  | Bayelsa | 69.3 | 55 |
|  | Benue | 22.9 | 24 |
|  | Borno | 63.9 | 23 |
|  | Cross River | 52.8 | 17 |
|  | Delta | 77 | 53 |
|  | Ebonyi | 46.2 | 7 |
|  | Edo | 88 | 50 |
|  | Ekiti | 82.1 | 26 |
|  | Enugu | 72.1 | 34 |
|  | FCT-Abuja | 69.6 | 80 |
|  | Gombe | 61.2 | 28 |
|  | Imo | 81.8 | 10 |
|  | Jigawa | 21.9 | 21 |
|  | Kaduna | 38.7 | 39 |
|  | Kano | 38.9 | 43 |
|  | Katsina | 33.2 | 26 |
|  | Kebbi | 30.3 | 36 |
|  | Kogi | 67.1 | 24 |
|  | Kwara | 51 | 50 |
|  | Lagos | 99.7 | 97 |
|  | Nasarawa | 20.3 | 32 |
|  | Niger | 37.4 | 35 |
|  | Ogun | 64.3 | 61 |
|  | Ondo | 57.5 | 28 |
|  | Osun | 80.9 | 50 |
|  | Oyo | 65.8 | 37 |
|  | Plateau | 17.1 | 31 |
|  | Rivers | 64 | 58 |
|  | Sokoto | 18 | 20 |
|  | Taraba | 17.4 | 17 |
|  | Yobe | 45.9 | 23 |
|  | Zamfara | 36.4 | 21 |
| Rwanda | City of Kigali | 71.4 | 74 |
|  | South | 11.3 | 13 |
|  | West | 20.4 | 14 |
|  | North | 16.8 | 12 |
|  | East | 20.1 | 13 |
| Senegal | Dakar | 97.7 | 98 |
|  | Diourbel | 59.8 | 65.3 |
|  | Fatick | 49.9 | 37.4 |
|  | Kaffrine | 21.1 | 22.1 |
|  | Kaolack | 46.2 | 47.4 |
|  | Kedougou | 33.5 | 18.2 |
|  | Kolda | 22.4 | 29.2 |
|  | Louga | 50.6 | 38.4 |
|  | Matam | 50.7 | 47.5 |
|  | Saint-Louis | 57.3 | 49.8 |
|  | Sedhiou | 30.6 | 24.4 |
|  | Tambacounda | 29.4 | 33.9 |
|  | Thies | 69.9 | 70 |
|  | Ziguinchor | 67.2 | 50.3 |
| Sierra Leone | Kailahun | 1.2 | 5.8 |
|  | Kenema | 12.7 | 17.9 |
|  | Kono | 15.5 | 24.6 |
|  | Bombali | 19.7 | 19 |
|  | Kambia | 2.2 | 6 |
|  | Koinadugu | 3.2 | 5.9 |
|  | Port Loko | 3 | 18.1 |
|  | Tonkolili | 6.7 | 7.9 |
|  | Bo | 27 | 28.1 |
|  | Bonthe | 5.2 | 7.1 |
|  | Moyamba | 2.7 | 3.9 |
|  | Pujehun | 1.4 | 4.5 |
|  | Western Rural | 20.2 | 65.9 |
|  | Western Urban | 91.3 | 81.4 |
| South Africa | Eastern Cape | 80 | 74 |
|  | Free State | 94.3 | 95 |
|  | Gauteng | 91.7 | 99 |
|  | KwaZulu Natal | 89.3 | 87 |
|  | Limpopo | 98.2 | 97 |
|  | Mpumalanga | 83.4 | 96 |
|  | North West | 92.6 | 95 |
|  | Northern Cape | 92.8 | 92 |
|  | Western Cape | 99.1 | 96 |
| Tanzania | Arusha | 27.6 | 43 |
|  | Dar es Salaam | 75.9 | 98 |
|  | Dodoma | 4.2 | 29 |
|  | Geita | 3.6 | 20 |
|  | Iringa | 27.6 | 37 |
|  | Kagera | 9.7 | 17 |
|  | Kaskazini Pemba | 22.9 | 48 |
|  | Kaskazini Unguja | 18.5 | 61 |
|  | Katavi | 7.1 | 15 |
|  | Kigoma | 8 | 17 |
|  | Kilimanjaro | 39.5 | 40 |
|  | Kusini Pemba | 28.6 | 44 |
|  | Kusini Unguja | 35.4 | 45 |
|  | Lindi | 8.8 | 21 |
|  | Manyara | 6.1 | 20 |
|  | Mara | 14 | 22 |
|  | Mbeya | 14.7 | 45 |
|  | Mjini Magharibi | 82.8 | 97 |
|  | Morogoro | 19.4 | 37 |
|  | Mtwara | 11.7 | 23 |
|  | Njombe | 17.5 | 22 |
|  | Pwani | 12.9 | 44 |
|  | Rukwa | 7 | 17 |
|  | Ruvuma | 13.2 | 21 |
|  | Shinyanga | 12 | 22 |
|  | Simiyu | 3.4 | 8 |
|  | Singida | 10.4 | 19 |
|  | Tanga | 29.6 | 35 |
| Togo | Centrale | 39.1 | 57 |
|  | Lomé | 93.8 | 99 |
|  | Kara | 28.9 | 46 |
|  | Maritime | 44.1 | 78 |
|  | Plateaux | 34.3 | 42 |
|  | Savanes | 15.1 | 31 |
| Zimbabwe | Manicaland | 15.6 | 30.5 |
|  | Mashonaland Central | 11.8 | 17.1 |
|  | Mashonaland East | 16.8 | 29.1 |
|  | Mashonaland West | 25.5 | 35.8 |
|  | Matabeleland North | 12.4 | 18.3 |
|  | Matabeleland South | 13.1 | 20.6 |
|  | Midlands | 24.3 | 31.8 |
|  | Masvingo | 20 | 20.5 |
|  | Harare Chitungwiza | 76.5 | 97.3 |
|  | Bulawayo | 96.3 | 97.1 |

## Figure 2.


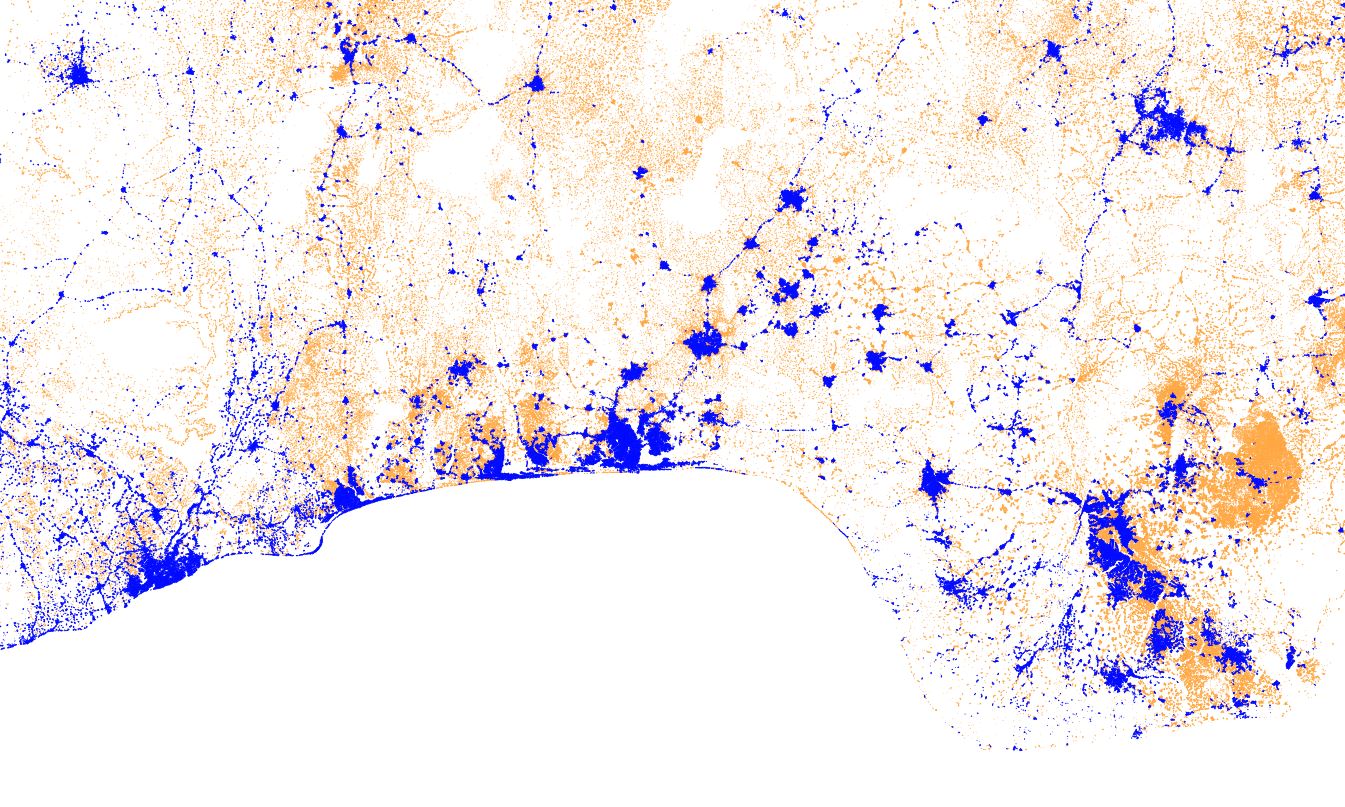


**Fig. 2.** Binary electrification status in coastal regions of Ghana, Togo, Benin and western Nigeria. Blue represents clusters with electricity accesses and yellow represents clusters that are not electrified.
